# Cigarette Smoking and Its Associations with Substance Use and HIV-Related Sexual Risks among Chinese Men Who Have Sex with Men

**DOI:** 10.3390/ijerph17051653

**Published:** 2020-03-05

**Authors:** Wenjian Xu, Wanjie Tang, Jing Zhang, Xin Shi, Yong Zheng, Michelle R. Kaufman

**Affiliations:** 1 Department of Sociology & Psychology, School of Public Administration, Sichuan University, Chengdu 610065, China; xuwenjian@scu.edu.cn; 2 Faculty of Psychology, Southwest University, Chongqing 400715, China; zhangjing17@email.swu.edu.cn (J.Z.); shixin9476@email.swu.edu.cn (X.S.); 3 Department of Health, Behavior & Society, Johns Hopkins University Bloomberg School of Public Health, Baltimore, MD 21205, USA; MichelleKaufman@jhu.edu; 4 Center for Educational and Health Psychology, Sichuan University, Chengdu 610065, China; tangwanjie2010@gmail.com; 5 Institute of Emergency Management and Post-disaster Reconstruction, Sichuan University, Chengdu 610065, China; 6 Mental Health Center, State Key Laboratory of Biotherapy, West China Hospital, Sichuan University, Chengdu 610065, China

**Keywords:** cigarette smoking, men who have sex with men, outness, homosexual stigma, substance use, HIV risk

## Abstract

China has the largest population of male smokers globally. Men who have sex with men (MSM) are at greater risk of cigarette smoking compared to the general population. Limited data are available regarding cigarette smoking and its associations with other health issues among Chinese MSM. Eligible MSM (n = 1100) were recruited from mainland China using an online national cross-sectional survey conducted in 2014–2015. Socio-demographic characteristics, smoking behavior, substance use, homosexual stigma and outness, HIV-related risk behavior, and HIV status were obtained. Logistic regression analyses were performed to evaluate predictors of current cigarette smoking frequency and the co-occurrence of smoking and drug use. Nearly 41% (n = 446) of participants had ever smoked cigarettes; 25% (n = 278) were current frequent smokers; and 13% (n = 138) were current infrequent smokers. Factors associated with a history of smoking included age, employment status, and monthly salary. Risk factors associated with current frequent smoking included self-identification as gay, having female sexual partners, binge drinking, drug use, higher levels of homosexual stigma, and being partially or fully “out” as gay. Adjusted multinomial analysis showed that human immunodeficiency virus (HIV) related risks, including risky sexual behaviors, lack of condom use, and a reported history of sexually transmitted infections (STIs), were associated with co-occurrence of current smoking and drug use. Cigarette smoking rates remain high among MSM in China. Cigarette smoking is associated with binge drinking, drug use, and HIV-related risks in this community. There is a clear opportunity for smoking cessation interventions to be linked with HIV and substance use prevention interventions, thereby addressing multiple health issues simultaneously for the MSM community in China.

## 1. Introduction

Cigarette smoking has been the second leading risk factor globally for early death and disability in recent years [1,2]. Smoking is the leading factor attributed to cardiovascular diseases, cancers, and chronic respiratory diseases, for both sexes [2]. These issues are highly relevant to the Chinese context. A study from 2010 demonstrated that over one third of cancer deaths in China were attributed to smoking [3]. Since official ratifications on the Framework Convention on Tobacco Control in 2005 and proceeding governmental programs and policies, the prevalence of cigarette smokers in China decreased substantially in the past 15 years, dropping by 22.4% and 48.4%, respectively, for males and females [2]. However, the vast majority of smokers in China are adult males [4]. As the country with the largest population in the world, China has an estimated 253 million male smokers, ranking first and accounting for one-third of global male smokers [2]. Given the substantial public health threats of smoking and the huge number of smokers, reducing tobacco use remains a major health challenge in China. Targeted interventions for communities and groups of adult males with high smoking rates, such as men who have sex with men (MSM), are needed.

### 1.1. Cigarette Smoking among MSM

Smoking disparities are related to sexual orientation [5]. Previous literature has demonstrated that MSM are at greater risk for cigarette smoking compared with general populations [6,7,8,9]. Although the rates of male cigarette smokers both in the United States (U.S.) and China have decreased, evidence suggests that MSM in China still smoke at high rates [6,7,10]. For example, one study that assessed smoking among MSM in Shanghai found that 65.9% were current smokers [10]. Possible explanations for these high rates are related to the experience of minority stress, as well as behaviors such as alcohol and illicit drug use [11]. Minority stress theory suggests that MSM experience a history of exclusion, discrimination, and victimization in many social settings, leading to daily experiences of stress as a result of their minority status [12,13]. Being an MSM is also associated with gender and sexuality-related adverse mental health outcomes, and tobacco is often used as a coping mechanism for managing minority stress [14]. Additionally, for social interaction, MSM may seek connections with other MSM in gay-identified bars and clubs, where high rates of smoking are present [12,13] and other unhealthy behaviors, such as alcohol consumption and illicit drug use, occur at significant levels [15]. These factors indicate a potential interaction and synergy between cigarette smoking and substance use. To better understand smoking mechanisms, it is of paramount importance to examine the co-occurrence of these behaviors.

### 1.2. Cigarette Smoking and HIV-Related Risks

MSM are among the groups with the highest human immunodeficiency virus (HIV) incidence and prevalence in many settings, including China [16,17]. In recent years, the rate of HIV infection among MSM in China has increased (from nearly 3.8% in 2003–2007 to 6.6% in 2013–2015) [18]. Unfortunately, the prevalence of smoking is high among HIV-positive MSM [19]. Smoking is related to increased incidence of HIV or acquired immunodeficiency syndrome (AIDS) related illnesses, malignancies, and mortality in HIV-positive MSM. Additionally, many studies have shown that MSM’s cigarette smoking is positively associated with HIV-related behavioral and sexual risks, including usage of alcohol and a variety of traditional and synthetic illicit drugs, such as heroin, marijuana, and methamphetamine [7,20,21], and a greater number of casual sex partners [20]. Meanwhile, sex with casual male partners and unprotected anal sex have been found to be associated with HIV and other sexually transmitted infections (STIs) [22,23,24]. A recent study conducted in the U.S. by the Multicenter AIDS Cohort Study found that smoking at least one pack of cigarettes per day was positively associated with being HIV-seropositive among MSM; furthermore, detectable viral load of HIV was significantly associated with prevalence of smoking among HIV-seropositive MSM [25]. One study documented that being HIV-positive was related to having ever attempted to quit smoking among Chinese MSM [19]. To our knowledge, few studies have examined the associations between cigarette smoking and HIV status among Chinese MSM.

### 1.3. Outness and Stigma

Additionally, unique factors for MSM related to sexual identity and individual level of stigma may contribute to risk for negative health behaviors including smoking [20]. Coming out, also known as “outness”, represents an important developmental stage for many gay individuals [26]. Studies in the U.S. and Mexico [20,27,28] show that gay men who conceal their homosexual identity are at elevated risk of other physical health problems (e.g., HIV progression and psychosomatic symptomatology), smoking, and other substance use. Outness may lead to resilience, as it contributes to feelings of self-acceptance and diminished internalized homophobia, increases the connection to potential positive social supports in the gay community, thereby reducing smoking behavior as a coping mechanism [11]. However, the process of coming out is complex, and it may be complicated or discouraged depending on the presence (or lack of) supportive cultural norms and homosexual stigma. In contrast to the U.S., China has not legally recognized same-sex marriages, and homosexuals still experience negative stereotyping, social stigma, and discrimination, contributing to the ability to be “out” [29]. The extent to which homosexuals expect to be stereotyped (anticipated stigma) could have important implications for how they experience their stereotyped status [30] and adjust or engage in behaviors to protect themselves from harm [5]. For instance, homosexuals might develop coping behaviors in opposition to dominant norms such as smoking [11,31,32], and even enter into heterosexual relationships and/or marriage with female partners as a form of “self-defense” against their homosexuality [33] and to comfort themselves.

### 1.4. The Present Study

There are limited data on cigarette smoking among MSM in China [10,19,34], including national Chinese MSM samples looking at this public health threat. Furthermore, Chinese MSM’s cigarette smoking, and its associations with risky behaviors such as binge drinking and drug use, unique individual factors such including stigma and outness, and HIV-related sexual behaviors (i.e., number of sexual partners and condom use), still remain unclear. Given MSM’s high HIV risk and the potential adverse effects of cigarette smoking and substance use to public health, the goal of this study was to: (1) explore the prevalence and patterns of cigarette use among Chinese MSM; (2) explore cigarette smoking in relation to HIV-related sexual risks; and (3) determine smoking and substance use associations with other variables and risk behaviors, using a national online convenience sample. Our objective was to identify points of intervention to address this and related public health issues among Chinese MSM.

## 2. Materials and Methods

### 2.1. Sampling Methods and Recruitment

As described in detail previously [35,36,37], a national sample of voluntary MSM participants were recruited from December 2014 to February 2015 using advertisements posted on social network platforms in China such as online gay chat rooms, popular gay dating apps (e.g., Blued and Zank), and instant messaging services (i.e., QQ groups and WeChat). Volunteers accessed the survey by clicking on the advertisement address. Those who clicked received a description of the study, and online informed consent was obtained from each participant. The study was conducted in accordance with the Declaration of Helsinki, and the protocol was approved by the Ethics Committee of the Cognitive and Personality Key Laboratory of Southwest University (Project Identification Code H14013). Upon completion, participants who provided an email address were entered into a raffle with a 6% chance of winning a 100 or 50 Chinese RMB (U.S.$16 or U.S.$8) prize. A total of 1,191 participants completed the survey. The eligible sample included in the analyses consisted of 1,100 MSM from 30 provinces and five regions of China (Central, East, South, West, North; presented in Table 1). Eligible participants were: (1) assigned male sex at birth; (2) a resident of mainland China; (3) aged 16 years or older; and (4) ever had sexual contact with other males. Participants who did not meet these criteria (n = 91) were excluded from the analysis.

### 2.2. Measures

#### 2.2.1. Socio-Demographic Characteristics

Participants were asked their gender, age, educational level, employment status, and monthly salary.

#### 2.2.2. Cigarette Smoking

Participants were asked if they had a history of cigarette smoking during their lifetime (yes/no). Additionally, participants were asked how often they had consumed cigarettes in the previous 6 months, for which the response options were: more than once per day, once per day, once per 2-to-3 days, once per week, once per 2 weeks, once per month, once per 2-to-3 months, once per 6 months, no, and unwilling to respond (6 participants). We categorized the MSM who reported consuming cigarettes at least once per day as current frequent smokers, MSM who reported consumption less than once per day as current infrequent smokers, and those who did not smoke in the previous 6 months as current non-smokers.

#### 2.2.3. Binge Drinking and Drug Use

For binge drinking, participants were asked how often they had 5 or more drinks of alcohol within 2 h [38] at any time in the previous 6 months (8 participants were unwilling to respond). This generated a dichotomous variable. For drug use, participants were asked if they had used rush poppers (alkyl nitrites), marijuana, crystal meth (methamphetamine), MDMA (ecstasy), ketamine, cocaine, or crack cocaine in the previous 6 months. Drug use was coded into a dichotomous variable to reflect at least one episode of using any type of drug.

#### 2.2.4. Sexual Identity, Attraction, and Role

Sexual identity was assessed using one question that asked participants to self-identify as gay, bisexual, heterosexual, or other. Sexual attraction, which included same-sex and opposite-sex attraction, was assessed with two separate questions: how sexually attracted is the participant to women, and how sexually attracted is the participant to men? Responses were given on a Likert scale of 1 (not attracted at all) to 5 (attracted very much). Anal sex role behavior was assessed using one question asking participants to report their role when engaging with a partner of the same sex, including insertive, receptive, and versatile.

#### 2.2.5. Sexual Behavior and Condom Use

Sexual behaviors were assessed by asking participants to report the number of male and female sexual partners they had over the previous 6 months. Condom use was assessed by asking participants to report the number of male and female sexual partners with which they did not use a condom in the previous 6 months.

#### 2.2.6. Perceived Homosexual Stigma and Outness

Perceived homosexual stigma was measured using the Stigma Consciousness Scale (e.g., “Stereotypes about homosexuals have not affected me personally”) adapted from Pinel [30]. This scale evaluates the notion of homosexual-related stereotype threat, or the feeling that occurs when situations instill in targets the fear of confirming stereotypes about the homosexual community. This scale is comprised of 10 items with a response set ranging from 1 (strongly disagree) to 7 (strongly agree). Items were reverse-coded and summed so that higher scores indicated increased expected homosexual discrimination and stigma consciousness. The scale was translated into Chinese and then back-translated by one English professional and two psychology graduates separately, until consensus was reached. Cronbach’s α for the scale in this study was 0.76.

Outness was measured with one question regarding the extent to which participants had disclosed their sexual orientation to others: never disclosed, partially disclosed, and fully disclosed.

#### 2.2.7. History of STIs and HIV Status

Participants were asked if they had ever had an STI diagnosis, including syphilis, human papillomavirus, gonorrhoea, genital herpes, and/or chlamydia. HIV status was assessed by asking participants to report their current HIV status, including HIV-negative, unsure of HIV status, and HIV-positive.

### 2.3. Statistical Analyses

SPSS 17.0 (IBM, Armonk, NY, USA) was used to characterize participants’ socio-demographics, sexual orientation, substance use, stigma and outness, and HIV-related risks. Descriptive analyses were conducted to assess the rate of cigarette smoking frequency within the previous 6 months. Pearson’s chi-squared test was performed to assess statistical differences by history and frequency of cigarette smoking. Latent profile analysis was performed to determine the number of homogenous groups or levels based on data from the Stigma Consciousness Scale using Mplus software. Univariable and adjusted multinomial logistic regressions—adjusted for confounding socio-demographic characteristics—were performed to evaluate the associations between frequency of current smoking and related factors. Multinomial logistic regression—adjusted for confounding socio-demographic characteristics—was performed to explore factors associated with the re-coded smoking and drug use variables. Variables were included within multinomial logistic regression models (including both frequency of current smoking and re-coded smoking and drug use) and were eliminated using a stepwise method until all correlates had a *p*-value < 0.10 [39]. For all logistic regression analyses, odds ratios (ORs) are reported with 95% confidence intervals (CIs).

## 3. Results

### 3.1. Characteristics of Participants

Among the 1100 completed surveys that were eligible for analyses, the mean participant age was 24.79 years (standard deviation = 6.10); 70.6% were educated at the college level or higher, 31.8% were students, and 61.2% were employed; 31.5% and 31.7% had a monthly salary between 2000 and 4000 Chinese RMB and more than 4000 Chinese RMB, respectively. Most MSM self-identified as gay (67.2%) and bisexual (21.7%; Table 1).

### 3.2. Cigarette Smoking, Sexual Orientation, Drug Use, Stigma, and HIV-Related Risks

Overall, 40.5% (n = 446) of MSM had a history of ever smoking cigarettes (Table 1); 38.0% reported cigarette smoking in the previous 6 months (n = 416). Furthermore, 12.6% (n = 138) were current infrequent smokers, and 25.4% (n = 278) were current frequent smokers (Table 2). Regarding other substance use, 44.3% had at least one binge drinking episode in the past 6 months, and 31.9% had used at least one drug. Among those who had a history of smoking, 44.2% (n = 188) had the co-occurrence of drug use. Among current smokers, 43.5% (n = 181) had co-occurrence of drug use. Among MSM, 58.4% had been with more than one male sexual partner, and 18.3% had been with female sexual partners, both in the previous 6 months. In the previous 6 months, 47.4% and 10.0% had had condomless sexual encounters with male and female partners, respectively. Nearly 10.2% ever had an STI diagnosis in their lifetime. Regarding HIV status, 13.2% were unsure, and 3.3% were HIV-positive.

Similar to analyses outlined in prior studies [40,41], latent profile analysis was performed for homosexual stigma. The plausibility of 2- to 5-class solutions were examined, and the 2-class solution was the best: Akaike information criterion = 40,744.81, adjusted Bayesian information criterion = 40,801.44, Entropy = 0.82, and Lo-Mendell-Rubin adjusted likelihood ratio test = 1034.22; *p*-value < 0.001. Thus, we created two levels: lower levels (n = 617) and higher levels (n = 483) of homosexual stigma.

### 3.3. Correlates of Cigarette Smoking

Among MSM, a history of cigarette smoking was associated with age (χ^2^ = 11.7; *p* < 0.01), employment status (χ^2^ = 40.4; *p* < 0.001), and monthly salary (χ^2^ = 26.4; *p* < 0.001). The frequency of current smoking was associated with age (χ^2^ = 25.3; *p* < 0.001), educational level (χ^2^ = 7.12; *p* < 0.05), employment status (χ^2^ = 62.5; *p* < 0.001), monthly salary (χ^2^ = 53.8; *p* < 0.001), and sexual identity (χ^2^ = 12.9; *p* < 0.05).

Adjusted multinomial logistic regressions (Table 3) demonstrated that self-identifying as gay significantly predicted frequent smoking (adjusted odds ratio (AOR) = 1.80, 95% CI: 1.02–3.19, *p* < 0.05). Compared to MSM without female sexual partners, those who had at least one female sexual partner were significantly more likely to be current frequent smokers (AOR = 2.23, 95% CI: 1.29–3.86, *p* < 0.01). Importantly, drug use (AOR = 1.66, 95% CI: 1.19–2.31, *p* < 0.01), binge drinking (AOR = 3.59, 95% CI: 2.62–4.92, *p* < 0.001), higher levels of homosexual stigma (AOR = 1.60, 95% CI: 1.17–2.19, *p* < 0.01), and partially or fully coming out (AOR = 1.59, 95% CI: 1.15–2.21, *p* < 0.01) had significantly higher odds of predicting frequent smoking. Despite a significant correlation between being HIV-positive and frequent smoking among MSM in the univariable analysis, this relationship disappeared in the multinomial analysis.

Failure to use a condom during sexual encounters with female partners (AOR = 1.51, 95% CI: 1.01–2.26, *p* < 0.05), drug use (AOR = 1.59, 95% CI: 1.05–2.43, *p* < 0.05), and binge drinking (AOR = 7.09, 95% CI: 4.59–11.0, *p* < 0.001) had significantly higher odds of predicting infrequent smoking.

### 3.4. Predictors of Smoking and Drug Use

Adjusted multinomial logistic regressions demonstrated that having multiple male sexual partners, having at least one female sexual partner, failure to use a condom during sexual encounters with male partners, and binge drinking were significantly more likely to occur within the current drug use only group and the co-occurrence of smoking and drug use group (details seen in Table 4) when compared to the neither current smoking nor drug use group. Additionally, higher levels of homosexual stigma (AOR = 1.65, 95% CI: 1.11–2.44, *p* < 0.01), partially or fully coming out (AOR = 2.00, 95% CI: 1.33–3.02, *p* < 0.01), and a reported history of STIs (AOR = 1.82, 95% CI: 1.01–3.29, *p* < 0.05) were significantly associated with co-occurrence of smoking and drug use.

## 4. Discussion

Due to the limited data currently available on cigarette smoking among MSM in China, we recruited an online national convenience sample, finding that the prevalence of a history of cigarette smoking and current smoking among MSM are high. Non-student status, self-identification as gay, higher levels of personal homosexual stigma, partially or fully coming out as gay, having at least one episode of sex with a female, binge drinking, and drug use were associated with current frequent smoking. Higher levels of homosexual stigma, partially or fully coming out, having multiple (at least 4) male sexual partners and condomless male sexual partners, having at least one female sexual partner, binge drinking, and a history of STIs were associated with co-occurrence of current smoking and drug use. There was no significant association between HIV status and cigarette smoking.

Our study contributes to the evidence that the rate of smoking among Chinese MSM remains high. The prevalence of a history of smoking in this study (40.5%) was lower than the rate of 65.9% found in a study conducted in 2008 in Shanghai, a small region of China, through respondent-driven sampling of MSM [10]. The lower prevalence of smoking in this online national sample might be due to increased tobacco control policies, such as increases in tobacco taxation, advertising bans, and smoke-free areas legislation in China over the past decade [2,42,43]. These policy-level interventions contribute to the reduction and prevention of tobacco use, and even reduce the likelihood of initiation of youth smoking [43]. Employment status may also affect the variety of smoking rates compared to the aforementioned study [10], where the sample contained nearly 50% male sex workers, who had a higher rate of smoking compared to the general MSM population. Nearly one third of MSM in our sample were students, who had a significantly lower rate of smoking compared to non-student MSM (including both employed and unemployed participants). Additionally, higher educational attainment may play a protective role in smoking behavior. With the recent popularization of higher education in China, the majority of our MSM sample was well educated (college level or higher; nearly 71%), and this rate is significantly higher than that in the aforementioned study (23.1%) [10].

This study assessed multiple components of sexual orientation (sexual identity, attraction, and behavior) and presented important information regarding the relationship between sexual orientation and tobacco use among Chinese MSM. Consistent with prior literature on MSM smokers [44], sexual identity was associated with current smoking; specifically, we found self-identified gay men were more likely to report current frequent smoking compared to heterosexual or other MSM. Inconsistent with one prior study [44], we did not find a relationship between sexual attraction and smoking, possibly due to variations in measurements and sample characteristics (no transgender women were included in the current study, but they were included in the Gerend study [44]. Regarding sexual behavior, having female (but not male) sexual partners was significantly associated with current frequent smoking after adjusting for potential confounding variables.

Minority stress has been found to be associated with tobacco use, binge drinking, drug use, stigma, and outness [11]. We found both binge drinking and drug use were associated with current smoking (including both infrequent and frequent smoking) among Chinese MSM, which is consistent with studies conducted in Western countries such as the U.S. [21,25] and one other study conducted in China [34]. This finding also suggests mechanisms of maladaptive behaviors, including cigarette smoking, binge drinking, and substance use, which all tended to emerge in a synergistic manner. Higher levels of homosexual stigma and coming out were associated with current frequent smoking. Traditional culture and a stigmatizing environment may complicate the process of outness and its associated health problems for Chinese MSM. As Pachankis and Hatzenbuehler have argued, experiences with sexual orientation-related stigma can lead homosexuals to engage in concealment behaviors [45,46], while coming out may make it difficult to have a positive self-identity and to practice healthy behaviors. Given the restrictive cultural and social norms of China, same-sex behavior may place individuals’ mental and/or physical health at risk. MSM who disclose their sexual identity may increase their exposure to minority stress and negative events; MSM who have higher levels of homosexual stigma may encounter more specific stigmatization, discrimination, and victimization, leading to increased rates of smoking.

Our findings provide important information from a collectivist culture perspective in that the results verify that cigarette smoking and drug use interact synergistically and co-occur with HIV-related risks such as multiple sexual partners, condomless sex, and STIs. In other words, MSM who have multiple male sexual partners and condomless sex with males were more likely to engage in the co-occurrence of smoking and drug use. Interestingly, having female sexual partners was also associated with the co-occurrence of smoking and drug use among MSM. Given the anti-homosexual social norms in China, some MSM feel pressured to have a relationship with or marry a woman [33,47]. Such stress may lead MSM in this environment to engage in substance use as a coping mechanism. Social cognitive theory [5,48], where behaviors are thought to be influenced by and learned from the social environment and their intrapersonal processing, may also explain multiple substance use among MSM. In addition, we found that MSM who reported a history of STIs were more likely to engage in the co-occurrence of smoking and drug use. Despite no relationship between smoking and HIV status in this study, previous research has demonstrated that risky behaviors and outcomes, such as having multiple male sexual partners, unprotected sexual intercourse, and STIs, are linked to a high prevalence of HIV infection among MSM [22,24]. In the current study, the online sampling methodology may not produce these same results.

### 4.1. Implications

There are several implications arising from this study. First, at the individual and community levels, smoking prevention and cessation interventions should be integrated with HIV prevention programs to sufficiently address factors affecting the health and well-being of Chinese MSM. In addition, such interventions should address substance use, unprotected sex, and minority stress as factors contributing to poor health. Second, at the health systems level, clinicians should take MSM’s cigarette and other substance use into consideration when performing an HIV test or general health assessment. Physicians could assist HIV-seropositive MSM patients with additional information regarding the adverse effects of cigarette smoking and substance use and link them to cessation and/or treatment programs. At the broader societal level, HIV prevention campaigns should include messages to reduce cigarette smoking, substance use, and HIV-related sexual behaviors, perhaps emphasizing holistic health messages for MSM.

### 4.2. Limitations

Some limitations should be noted in interpreting the current findings. First, causal effects might not be determined due to the data being cross-sectional. Second, we utilized an online sampling method, which does not allow us to determine the response rate or the characteristics of those who chose not to participate. As a result, these findings may not be generalizable to the MSM population as a whole. Also, online survey methods are more likely to result in younger MSM samples. Third, the present study did not compare the behaviors of MSM with sexual majority males, which precludes the ability to know whether smoking and health disparities are influenced by sexual identity. Fourth, tobacco cessation efforts were not assessed, nor was the use of e-cigarettes or vaping, as use of such products is low among youth in China (only 1.2% report use in the past month) [49]. Fifth, STI self-report data may not be accurate. Finally, measurements and reports of outness, sexual behaviors, and some drug use that is illicit in China may result in biased responses—some participants who are sensitive to discussing such issues as a result of cultural norms and social desirability may not be as forthcoming in reporting their behaviors [50]. Future research should include longitudinal samples and examine predictors and consequences of cigarette use to further explore findings for this population.

## 5. Conclusions

This study provides important information regarding the high prevalence of both a history of and current cigarette smoking in an online national Chinese MSM sample. This study verifies the associations between cigarette smoking and substance use and HIV-related risks from a cross-cultural perspective. This study also presents a potential disparity in cigarette smoking by sexual orientation in China. These results may be valuable in guiding interventions targeting this marginalised Chinese community to address smoking, other substance use, and HIV risk behavior.

## Figures and Tables

**Table 1:** Participant socio-demographic characteristics and sexual identity by lifetime history of smoking among a national sample of Chinese men who have sex with men (MSM).

	Lifetime History of Smoking			
	Total (1100; %)	No (654; 59.5%)	Yes (446; 40.5%)	Statistics (*p*)
**Socio-demographic characteristics**				
Region of China				3.44 (0.487)
Central	256 (23.3)	144 (22.0)	112 (25.1)	
East	208 (18.9)	134 (20.5)	74 (16.6)	
South	125 (11.4)	76 (11.6)	49 (11.0)	
West	427 (38.8)	251 (38.4)	176 (39.5)	
North	84 (7.6)	49 (7.5)	35 (7.8)	
Age				11.7 (0.001)
16–25 years	640 (58.2)	408 (62.4)	232 (52.0)	
>25 years	460 (41.8)	246 (37.6)	214 (48.0)	
Educational level				1.49 (0.223)
High school or less	323 (29.4)	183 (28.0)	140 (31.4)	
College or more	777 (70.6)	471 (72.0)	306 (68.6)	
Employment status				40.4 (<0.001)
Student	350 (31.8)	255 (39.0)	95 (21.3)	
Employed	673 (61.2)	364 (55.7)	309 (69.3)	
Unemployed (Non-student)	77 (7.0)	35 (5.4)	42 (9.4)	
Monthly salary				26.4 (<0.001)
<¥2000	405 (36.8)	281 (43.0)	124 (27.8)	
¥2000–¥4000	346 (31.5)	183 (28.0)	163 (36.5)	
≥¥4000	349 (31.7)	190 (29.1)	159 (35.7)	
**Sexual identity**				3.66 (0.160)
Gay	739 (67.2)	429 (65.6)	310 (69.5)	
Bisexual	239 (21.7)	143 (21.9)	96 (21.5)	
Heterosexual/Other	122 (11.1)	82 (12.5)	40 (9.0)	

**Table 2:** Participant socio-demographic characteristics and sexual identity by frequency of current smoking among a national sample of Chinese MSM.

		Frequency of Smoking			
	All (*n* = 1094)	No (678; 62.0%)	Infrequent (138; 12.6%)	Frequent (278; 25.4%)	Statistics (*p*)
**Socio-demographic characteristics**					
Region of China					11.76 (0.162)
Central	253 (23.1)	141 (20.8)	36 (26.1)	76 (27.3)	
East	208 (19.0)	143 (21.1)	26 (18.8)	39 (14.0)	
South	124 (11.3)	78 (11.5)	11 (8.0)	35 (12.6)	
West	425 (38.8)	264 (38.9)	56 (40.6)	105 (37.8)	
North	84 (7.7)	52 (7.7)	9 (6.5)	23 (8.3)	
Age					25.3 (<0.001)
18–25 years	639 (58.4)	429 (63.3)	83 (60.1)	127 (45.7)	
>25 years	455 (41.6)	249 (36.7)	55 (39.9)	151 (54.3)	
Educational level					7.12 (0.028)
High school or less	318 (29.1)	185 (27.3)	35 (25.4)	98 (35.3)	
College or more	776 (70.9)	493 (72.7)	103 (74.6)	180 (64.7)	
Employment status					62.5 (<0.001)
Student	347 (31.7)	269 (39.7)	39 (28.3)	39 (14.0)	
Employed	670 (61.2)	369 (54.4)	91 (65.9)	210 (75.5)	
Unemployed (Non-student)	77 (7.0)	40 (5.9)	8 (5.8)	29 (10.4)	
Monthly salary					53.8 (<0.001)
<¥2000	403 (36.8)	302 (44.5)	43 (31.2)	58 (20.9)	
¥2000–¥4000	343 (31.4)	194 (28.6)	51 (37.0)	98 (35.3)	
≥¥4000	348 (31.8)	182 (26.8)	44 (31.9)	122 (43.9)	
**Sexual identity**					12.9 (0.012)
Gay	736 (67.3)	441 (65.0)	86 (62.3)	209 (75.2)	
Bisexual	237 (21.7)	151 (22.3)	37 (26.8)	49 (17.6)	
Heterosexual/Other	121 (11.1)	86 (12.7)	15 (10.9)	20 (7.2)	

**Table 3:** Univariable and multinomial analyses: factors associated with current smoking with the current non-smokers group as reference.

	Current Infrequent Smoking		Current Frequent Smoking	
	OR (95% CI)	AOR (95% CI)	OR (95% CI)	AOR (95% CI)
Socio-demographic characteristics				
Age (>25 years)	1.14 (0.78,1.66)	-	2.05 (1.54,2.72) ***	-
Educational level (College or more)	1.10 (0.73,1.68)	-	0.69 (0.51,0.93) *	-
Employment status (Ref. Student)				
Employed	1.70 (1.13,2.56) *	1.79 (1.13,2.82) *	3.93 (2.70,5.72) ***	3.33 (2.20,5.03) ***
Unemployed (Non-student)	1.38 (0.60,3.16)	1.56 (0.65,3.76)	5.00 (2.79,8.97) ***	4.75 (2.53,8.91) ***
Monthly salary (Ref. < ¥2000)				
¥2000–¥4000	1.85 (1.18,2.88) **	-	2.63 (1.81,3.81) ***	-
≥¥4000	1.70 (1.08,2.69) *	-	3.49 (2.43,5.02) ***	-
Sexual identify (Ref. Heterosexual/Other)				
Gay	1.12 (0.62,2.03)	0.86 (0.45,1.65)	2.04 (1.22,3.41) **	1.80 (1.02,3.19) *
Bisexual	1.41 (0.73,2.71)	1.07 (0.53,2.18)	1.40 (1.22,3.41)	0.95 (0.50,1.81)
Attraction to males *M* (*SD*)	0.93 (0.72,1.20)		1.06 (0.86,1.30)	
Attraction to females *M* (*SD*)	1.01 (0.85,1.21)		0.88 (0.77,1.01)	
Anal sex role (Ref. Insertive preference)				
Receptive preference	0.62 (0.40,0.93) *	0.70 (0.44,1.11)	0.69 (0.50,0.95) *	0.84 (0.58,1.23)
Versatile	0.65 (0.35,1.20)	0.66 (0.34,1.29)	0.53 (0.32,0.89) *	0.62 (0.35,1.09)
Sexual behavior				
Number of male partners (2–3)	1.44 (0.91,2.27)	-	1.16 (0.81,2.67)	-
Number of male partners (≥4)	1.84 (1.18,2.85) **	-	1.92 (1.38,2.67) ***	-
Number of female partners (≥1)	1.28 (0.78,2.08)	1.51 (0.77,2.98)	2.36 (1.68,3.32) ***	2.23 (1.29,3.86) **
Condom use				
Condomless male partners (≥1)	1.55 (1.07,2.23) *	1.51 (1.01,2.26) *	1.35 (1.02,1.79) *	1.12 (0.81,1.54)
Condomless female partners (≥1)	0.71 (0.33,1.53)	0.31 (0.12,0.84) *	2.29 (1.50,3.49) ***	1.11 (0.58,2.11)
Drug use (Yes)	2.24 (1.53,3.27) ***	1.59 (1.05,2.43) *	2.45 (1.82,3.29) ***	1.66 (1.19,2.31) **
Binge drinking (Yes)	6.99 (4.58,10.7) ***	7.09 (4.59,11.0) ***	3.41 (2.55,4.56) ***	3.59 (2.62,4.92) ***
Homosexual stigma (Higher score)	1.42 (0.98,2.05)	1.31 (0.88,1.94)	1.76 (1.33,2.34) ***	1.60 (1.17,2.19) **
Outness (Partially/Fully)	1.29 (0.89,1.89)	1.26 (0.82,1.92)	1.65 (1.24,2.19) **	1.59 (1.15,2.21) **
History of STIs (Yes)	1.66 (0.93,2.96)	-	2.04 (1.32,3.15) **	-
HIV status (Ref. Negative)				
Unsure	1.06 (0.63,1.78)	-	0.89 (0.58,1.37)	-
Positive	0.27 (0.04,2.04)	-	2.35 (1.19,4.65) *	-

Note: OR = odds ratio; AOR = adjusted odds ratio; CI = confidence interval. * *p* < 0.05; ** *p* < 0.01; *** *p* < 0.001. Adjusted for potential confounding socio-demographic characteristics, including age, educational level, employment status, and monthly salary.

**Table 4:** Adjusted multinomial analyses (as MSM with neither current smoking nor drug use as reference); factors associated with cigarette smoking and drug use.

	Current Smoking Only	Current Drug Use Only	Co-Occurrence of Smoking and Drug Use
	AOR (95% CI)	AOR (95% CI)	AOR (95% CI)
Socio-demographic characteristics			
Educational level (College or more)	0.83 (0.57,1.21)	1.48 (0.95,2.31)	1.23 (0.78,1.92)
Employment status (Ref. Student)			
Employed	2.35 (1.20,4.63) *	1.65 (0.74,3.66)	2.97 (1.32,6.65) **
Unemployed (Non-student)	3.03 (1.49,6.15) **	1.49 (0.62,3.55)	4.20 (1.75,10.1) **
Monthly salary (Ref. < ¥2000)			
¥2000–¥4000	1.25 (0.66,2.36)	1.46 (0.68,3.15)	1.64 (0.77,3.50)
≥¥4000	1.35 (0.68,2.67)	2.06 (0.92,4.59)	2.44 (1.10,5.38) *
Anal sex role (Ref. Insertive preference)			
Receptive preference	0.99 (0.66,1.49)	1.92 (1.18,3.14) **	1.23 (0.77,1.96)
Versatile	0.75 (0.41,1.35)	1.60 (0.83,3.10)	0.83 (0.42,1.67)
Sexual behavior			
Number of male partners (2–3)	1.28 (0.85,1.94)	2.69 (1.66,4.35) ***	1.31 (0.76,2.23)
Number of male partners (≥4)	1.19 (0.75,1.87)	3.19 (1.93,5.28) ***	3.31 (2.00,5.48) ***
Number of female partners (≥1)	2.03 (1.09,3.78) *	2.18 (1.10,4.33) *	2.84 (1.45,5.54) **
Condom use			
Condomless male partners (≥1)	1.24 (0.86,1.78)	1.71 (1.15,2.55) **	1.85 (1.21,2.82) **
Condomless female partners (≥1)	0.52 (0.24,1.13)	0.33 (0.13,0.83) *	0.54 (0.24,1.22)
Binge drinking (Yes)	4.80 (3.39,6.79) ***	1.65 (1.11,2.44) **	6.29 (4.16,9.49) ***
Homosexual stigma (Higher score)	1.43 (1.02,2.00) *	0.98 (0.67,1.43)	1.65 (1.11,2.44) **
Outness (Partially/Fully)	1.35 (0.95,1.93)	1.12 (0.75,1.67)	2.00 (1.33,3.02) **
History of STIs (Yes)	1.06 (0.57,1.96)	1.25 (0.67,2.35)	1.82 (1.01,3.29) *

Note: AOR = adjusted odds ratio; CI = Confidence interval. * *p* < 0.05; ** *p* < 0.01; *** *p* < 0.001. Adjusted for potential confounding socio-demographic characteristics, including educational level, employment status, and monthly salary.

## Abbreviations

AIDS	acquired immunodeficiency syndrome
AOR	adjusted odds ratio
CI	confidence interval
HIV	human immunodeficiency virus
MSM	Men who have sex with men
STIs	sexually transmitted infections
U.S.	United States
